# The elephant in the room: reflecting on text-to-image generative AI and global health images

**DOI:** 10.1136/bmjgh-2024-015601

**Published:** 2024-04-08

**Authors:** Arsenii Alenichev, Patricia Kingori, Jonathan Shaffer, Koen Peeters Grietens

**Affiliations:** 1 Nuffield Department of Population Health, University of Oxford, Oxford, UK; 2 Department of Population Health, University of Oxford, Oxford, UK; 3 Sociology, University of Vermont, Burlington, Vermont, USA; 4 Instituut voor Tropische Geneeskunde, Antwerpen, Belgium

**Keywords:** public health, accountability

There has been increasing evidence that generative AI produces biased, exaggerated and otherwise problematic images with regard to class, race and gender, among other socially enacted markers.^1 2^ Further extending these concerns to global health and text-to-image generation,^3^ in this commentary article we discuss ‘the elephant in the room’ both as a metaphor for global health,^4^ visual culture and stereotypical depictions of ‘Africa’, and in the literal sense, as shown in figure 1.

**Figure 1:**
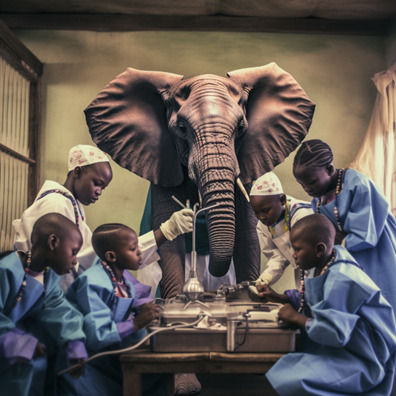
The Elephant in the room. This image was created from a ‘doctors help children in Africa’ prompt in Midjourney V.5.1.

Generative AI does not exist in a vacuum. Each generative AI platform is trained (presumably, since their methods and datasets are notoriously opaque) on the work of thousands of photographers, artists and millions of images. While AI’s rendering of an elephant as a doctor supervising children might be shocking at first glance, such visual stereotypes are not new. For decades, scholars and activists have been tracing ways in which Western texts and images depicted ‘Africa’ through the racialised lens of the colonial ‘other’,^5^ resulting in a number of negative and stereotyped ideas, such as Africa as an accumulation of misery, as an uncivilised dangerous space requiring external moderation, and a complete opposite: a vast savannah in which people and animals live in harmony.^6–8^ Many of these imaginaries of Africa have been perpetuated and even invigorated by the entertainment industry—particularly via movies and cartoons^9^—leading to what is sometimes called the ‘disneyfication’ of the continent with prime examples being *The Lion King*, *Tarzan* or the Madagascar series. A recent analysis of 50 movies produced over a span of 100 years showed three main tropes of how the continent has been portrayed: ‘the wild Africa’ without urban settlements; ‘the exotic colonial city’, emphasising the street markets and westerners encountering the mysterious ‘other’; and, ‘the Afropessimist city’ underlining urban poverty, corruption and violence.^10^

In the era of neoliberal globalisation, stereotypes of Africa as wildlife, savannah and unspoiled communities are further fuelled by the tourist industry’s countless websites, texts and images, targeting wealthy outsiders who can now experience handpicked mise-en-scènes that fit the western hallucination about the continent.^11^ A telling example was the 2014 image of a giraffe tweeted by Delta Airlines to depict Ghana; there are no giraffes in Ghana.^12^ The sheer magnitude of such imaginations and the reified power of postcolonial political economy shaping this imagery has led some African organisations themselves to re-enact a stereotypical ‘Africa’. Sometimes theorised as autoexoticism,^13^ such process involves a rational and purposeful local invocation of stereotypes in response to the desires of tourists and other international stakeholders, as evident, for instance, in ethnographies of how members of pastoral communities actively enact themselves as ‘unspoiled communities’ to be visited.^14^

Moving from stereotypical tranquillity to ‘misery’ and ‘incapacity’, the humanitarian industry and its colonial predecessors have long rendered a rubric involving both the stereotype of a ‘suffering African child’ and the international care and relief provided courtesy of an organisation and altruistic volunteers, often encapsulated as ‘white saviour’^15^ images, also reproduced by AI (figure 2). One influential cultural reflection on this topic has been formed by the famous Humanitarians of Tinder blog, offering a collection of screenshots of people using humanitarian white saviour images as their profile pictures for the popular dating app.

**Figure 2:**
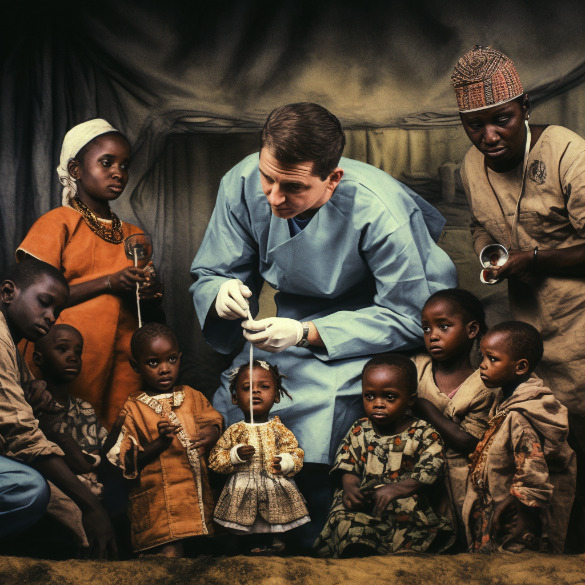
'Doctors help children in Africa'. Midjounrey 5.1.

Taking an active stance against such stereotypes of Africa, many artists, activists, thinkers and scholars actively work to depict the continent and its 55 countries differently, such as in African modernity and African futurism. However, despite these intentions, the sheer magnitude of stereotypical visual signifiers is overwhelming, dazzling and deafening: suffering subjects, wildlife, wooden masks, volunteers, unspoiled communities and misery. As an exercise, we did a challenge to find those visual stereotypes as fast as possible: it took us less than a minute of using an internet search engine to encounter a popular volunteer website advertising visiting ‘rural villages’ in Kenya and helping the locals, or interacting—yes you got it—with the elephants as part of the ‘real Ghana experience’.

While it is not exactly clear how different generative AI tools decompose and tokenise real image training data for reconstruction in response to text prompts, it does so nonetheless. Many of these visuals and conceptions about the continent become a vast visual substrate for various AI tools to ‘learn’ from, produce significations of ‘Africa’ and, recursively reproduce images on demand, as evident from figures 1 and 2. Is it surprising that AI, by absorbing millions of biased and stereotypical ‘real’ images of Africa, is producing something similar as a result? In the same way abusive and biased depictions of ‘Africa’ have been populating the internet for decades, an elephant in the room is perhaps not merely a ‘glitch in the system’, but in fact a rational and predictable product of centuries of coloniality, othering, misrepresentation and lazy assumption.

A transdisciplinary global health inquiry into the relationship between real and AI-generated images and stereotypes is especially relevant right now; with the unprecedented international rollout and use of generative AI, it is unsurprising that global health organisations are attracted to using AI-generated images. Compared with standard photography conducted in person, AI-generated imagery is often considered practically, economically and ethically advantageous. For instance, it can allow organisations to protect the anonymity of vulnerable people and communities. AI images are argued to be more environmentally friendly as photographers do not need to travel. And, even more pragmatically, AI enables organisations to save money and time. For instance, a photographer’s labour for a global health photoshoot can cost thousands of dollars and it requires several days to organise the logistics, permits and arrangements for participants. An AI image requires far fewer resources and it can be created in minutes. Moreover, no informed consent is required from the photography subjects and there are no copyright negotiations with photographers. These factors are among some of the reasons why organisations such as Amnesty International, Plan International, Charity Right, and the WHO already have a record of using AI-generated images for marketing and communication purposes.

The strategic creation and dissemination of images to depict people and communities is a cheap way of achieving political objectives in international relations.^16^ Images, therefore, are not simply representations but active political agents that help construct relationships of difference and frame political struggle. This simple point leads to a difficult question: What are we to do with this elephant in the room of global heath visual culture, and the relationship between ‘real’ stereotypes and their proliferation via AI? There is of course a solution offered by AI ethics and safety—that is, to simply fix the imperfect datasets and biases. However, we believe that the technical fix of moderating datasets and algorithms will only offer a short-term ‘comforting’ solution, overlooking—or even hiding—the elephant in the room: millions of real images and shared histories marked by abuse and exploitation. Ultimately, the definitive solution would be the emergence of societies wherein both real and AI-generated global health images will not be needed for the effective functioning of healthcare systems, while investing into genuine bottom-up photojournalism and supporting local representational efforts radically departing from the omnipresent colonial visualisations of Africa and other colonially misrepresented spaces. The end goal should be to create the conditions in which the legacy of abusive global health depictions will become so detached from material realities that they eventually become obsolete.

At long last, may the elephant be set free.

## Data Availability

All data relevant to the study are included in the article.
